# Down-Regulation of *FAD2-1* Gene Expression Alters Lysophospholipid Composition in the Endosperm of Rice Grain and Influences Starch Properties

**DOI:** 10.3390/foods10061169

**Published:** 2021-05-23

**Authors:** Jixun Luo, Lei Liu, Christine Konik-Rose, Lijun Tian, Surinder Singh, Crispin A. Howitt, Zhongyi Li, Qing Liu

**Affiliations:** 1 Commonwealth Scientific and Industrial Research Organisation, Agriculture & Food, Acton, ACT 2601, Australia; Christine.Konik-Rose@csiro.au (C.K.-R.); Lijun.Tian@csiro.au (L.T.); Surinder.Singh@csiro.au (S.S.); Crispin.Howitt@csiro.au (C.A.H.); Zhongyi.Li@csiro.au (Z.L.); Qing.liu@csiro.au (Q.L.); 2 Commonwealth Scientific and Industrial Research Organisation, Precision Health Future Science Platform, Acton, ACT 2601, Australia; 3 Southern Cross Plant Sciences, Faculty of Science and Engineering, Southern Cross University, Lismore, NSW 2480, Australia; Ben.liu@scu.edu.au

**Keywords:** high oleic rice, fatty acid, starch, amylose-lipid complex, lysophospolipid, phospholipid

## Abstract

Small quantities of lipids accumulate in the white rice grains. These are grouped into non-starch lipid and starch lipid fractions that affect starch properties through association with starch. Lysophosphatidylcholine (LPC) and lysophosphatidylethanolamine (LPE) are two major lipid classes in the two fractions. Using high-oleic rice grains, we investigated the fatty-acid composition in flour and starch by LC-MS and evaluated its impact on starch properties. In the wild-type grain, nearly 50% of fatty acids in LPC and LPE were palmitic acid (C16:0), over 20% linoleic acid (C18:2) and less than 10% oleic acid (C18:1). In the high-oleic rice grain, C18:1 increased at the expense of C18:2 and C16:0. The compositional changes in starch lipids suggest that LPC and LPE are transported to an amyloplast with an origin from endoplasmic reticulum-derived PC and PE during endosperm development. The high-dissociation temperature of the amylose-lipid complex (ALC) and restricted starch swelling power in the high-oleic rice starch indicates that the stability of the ALC involving C18:1 is higher than that of C18:2 and C16:0. This study provides insight into the lipid deposition and starch properties of rice grains with optimized fatty-acid composition.

## 1. Introduction

Lipids comprise 1–4% of whole grain rice and most of it is concentrated in the bran and embryo while a small amount exists in the starch-rich endosperm. Total lipids extracted from rice bran consist of 84.9–86.0% triacylglycerols (TAGs), 6.5–6.7% phospholipids (PLs) and 4.2–4.6% free fatty acids (FFAs) [1]. The lipids found in the endosperm can be grouped into starch lipids and non-starch lipids, with the former located inside starch granules and the latter attached to their surface [2,3]. Non-starch lipids are mainly TAGs, PLs, and glycolipids, while starch lipids are mostly lysophospholipids (LPL), mainly lysophosphatidylcholine (LPC) and lysophosphatidylethanolamine (LPE) in addition to FFA [3,4]. LPC and LPE comprise up to 50% of the starch lipid [5].

Although lipids are minor components of the rice endosperm, they play an important role in starch quality through their combination and interaction with the major grain component, starch, which is synthesized mainly through glucose polymerizing and forms granules in the amyloplast of the endosperm cells during seed development. The lipid content of white rice grain is generally correlated with the amylose content of starch, especially the starch lipids that are believed to be physically associated with the amylose molecules [6]. Amylose is a component of starch with a linear long-chain polysaccharide made of α(1,4)-glucose units with sparse branches linked by the α(1,6)-glucose bonds. The native amylose molecular structure is a single left-handed helix with a hydrophobic cavity and hydrophilic surface, which can form a supramolecular amylose-lipid complex (ALC) [7,8]. The intermolecular forces, such as van der Waals force and hydrogen bonding, can stabilize the amylose-lipid interaction. One turn of an amylose helix consists of six or eight glucose units, and it is suggested that 18–24 glucose units can hold one molecule of an FFA or a monoacyl lipid molecule [7,8].

The formation of ALCs restricts the swelling of starch granules, increases gelatinization temperature, reduces starch hydrolysis and significantly affects starch properties and digestibility in both food and animal feed [7,9,10]. The ALC properties are influenced by the chain length of amylose, and the content and profiles of the lipids. For example, the long amylose chain facilitates the formation of a stable ALC, accommodates lipid molecules and results in high dissociation temperatures [11,12]. It is suggested that a 0.3% (*w/w*) lipid can induce ALC formation, and a 10% lipid can theoretically complex with all the amylose [7]. The crystallinity of V-type starch formed by fatty acids reduces as the length of fatty acids increases [13]. The other starch component, amylopectin, is a highly branched glucan polymer produced by the formation of frequent α(1,6)-glucose bonds between the linear glucan chains. In contrast to amylose, the ability of amylopectin to interact with lipids is rather limited due to its semicrystalline structure with short α(1,6)-linked glucan branches and large steric effects [13,14].

Through intermolecular interactions, LPLs make major contributions to the functionality of ALCs. In this context, much work has been done on the lipids of cereal grain endosperms [4,15,16,17]. It is generally believed that the properties of LPLs and their constituent fatty acids are attributable to the stability and range of physicochemical properties of ALCs [7]. For example, starch viscosity and swelling property and the digestibility of an ALC in wheat and rice are affected by the composition of LPLs [18]. The length of a fatty acid molecule is positively correlated to the melting temperature of the ALC [19,20]. The double bonds result in bends or kinks in the conformation of unsaturated fatty acids, which can influence the capacity of the fatty acids to complex with amylose [21,22,23]. However, only a few research works have been devoted to the fatty acid composition of starch lipids, so the relationship between the change of starch fatty acid composition and the quality of native starch in rice grains remains unclear.

Plant fatty acid de novo biosynthesis occurs in the plastid. The synthesised fatty acids are incorporated into plastidial lipids including galactolipids and phospholipids via the prokaryotic pathway. In addition, de novo synthesized fatty acids can enter extraplastidial membrane lipids including phosphatidylcholine (PC) via an acyl editing process or by the conversion of diacylglycerol (DAG), which is synthesised in the endoplasmic reticulum (ER) to PC. DAG backbones are the precursors both for plastid lipid synthesis and eukaryotic phospholipids PC and phosphatidylethanolamine (PE) [24]. Due to the different fatty-acid specificity of the *sn*-2 acyltransferases, a C16 fatty acid at the *sn*-2 position is a signature for chloroplastidial lipids, while the ER-derived phospholipids typically carry a C18 fatty acid at the *sn*-2 position. Eukaryotic phospholipid PC is synthesised in the ER but is also present in the outer chloroplast envelope [25].

Further desaturation from C18:1 to C18:2 at the *sn*-2 position is catalysed by fatty acid desaturase 2 (FAD2). We have previously reported on the genetic down-regulation of the major *FAD2* gene (*FAD2-1*) in the seed via RNA interference (RNAi) [26]. The C18:1 content nearly doubled in the total lipids of rice grains at the expense of C18:2 and C16:0 [26]. Acyl-CoA:lysophosphatidylcholine acyltransferase (LPCAT) enzymes play a central role in acyl editing of PC via the deacylation of PC to lysophosphatidylcholine and acylation of LPC to PC. To our knowledge, the potential impact of the increased ratio of C18:1 to C18:2 on the structure and functional properties of an ALC remains unexplored. Since lysophospholipids especially LPC and lysophosphatidylethanolamine are the dominant starch lipids and play an important role in starch quality [3,4,5], in this study, high oleic rice grains resulting from *FAD2*-*1* down-regulation (*FAD2^DR^*) were used to investigate starch lipids deposition in the endosperm and characterise starch functional properties.

## 2. Materials and Methods

### 2.1. Developing FAD2^DR^ Backcross Population and Genotyping by PCR

For the study, a cross was made between the previously developed *FAD2*-*1* RNAi line and its background variety *O. sativa* cv. Nipponbare to create a population with a clear genetic background [26]. The F_1_ seed of the *FAD2*-*1* RNAi line backcross was grown to develop the F_2_ population for DNA marker-assisted selection up to the F_4_ generation. All plants were grown in the phytotron at CSIRO Black Mountain Scientific Innovation Park (Canberra, ACT, Australia) at 23–29 °C under natural daylight. Leaf tissue was collected from each plant from generations F_1_ to F_4_ for quick DNA extraction [27]. DNA markers were used to identify the progeny carrying the *FAD2-1* RNAi constructs by PCR as reported previously [26]. Mature seeds were harvested and manually threshed and dehulled to produce brown rice grains for the following analysis.

### 2.2. Phenotyping by Total Fatty Acid Profiling Using Gas Chromatography

For screening purposes, 12 brown rice grains were selected from each F_2_ line carrying the RNAi construct for total fatty acid (TFA) profiling. Each grain was loaded into a 2 mL Eppendorf tube, and ground into flour using a 3M ESPE CapMax™ homogenizer (ESPE, Seefeld, Germany). About 4 mg of flour was weighed for total lipid extraction and fatty acid methyl ester (FAME) preparation following the published procedure with modification as described by Zaplin et al. [26], and analyzed by gas chromatography (7890A GC; Agilent Technologies, Santa Clara, CA) fitted with an SGE BPX70 column (0.25 mm diameter, 60 m length, 0.25 µm film thickness) and helium as the carrier gas. Raw data were integrated and analyzed using Agilent Technologies ChemStation software (Rev B.04.03). The composition of each fatty acid was calculated as the percentage of the TFA. Three *FAD2-1* RNAi (*FAD2^DR^*) segregants in F_2_ progeny and three wild-type *FAD2-1* (*FAD2^WT^*) segregants were selected as the control for the following analysis. Phenotyping by single grain TFA profiling was repeated using 12 grains of the selected lines to confirm genetic stability until F_4_.

### 2.3. Rice Grain Polishing and Starch Extraction

Brown rice grains of 150–200 mg of each selected line were loaded into a homemade polisher fitted with an abrasive metal brush and sandpaper. Rice bran was removed from the brown rice grains by polishing for 1 min to produce white rice grains. These were then ground in a capsule with ball bearings using a 3M ESPE CapMix™ capsule mixer (ESPE, Seefeld, Germany) to produce white rice flour, of which approximately 500 mg of grains from each line were washed with 0.005% NaOH and filtered through 0.5 mm nylon sieves followed by centrifugation at 10,000× *g* for 2 min. The pellet was washed with water and resuspended in a 50 mM phosphate buffer (pH 7.5) containing 50 µg/mL proteinase K. After incubation for 2 h, the obtained starch extract was rewashed with water three times. The starch pellet collected by centrifugation was freeze-dried overnight.

### 2.4. Quantitative Analysis of Lysophospholipids Using Liquid Chromatography−Mass Spectrometry

Total lipids were extracted from white rice grain and LPLs were determined in duplicates for all three biological replicates by a Liquid Chromatography−Mass Spectrometry (LC−MS) method described by Liu et al. [28]. Briefly, non-starch lipids were obtained by exhaustively extracting approximately 15 mg of rice flour with chloroform/methanol (2:1, *v/v*; 0.5 mL) three times sequentially. The liquid extracts were combined and dried under a nitrogen stream. The dried extracts were dissolved in 0.8 mL of chloroform/methanol (2:1, *v/v*) and the non-starch LPLs were quantified by LC−MS according to Liu et al. [28]. Included in the quantification were the LPC 18:0 (*m/z* 524, eluted at 6.4 min) and LPE 18:0 (*m/z* 482, eluted at 6.0 min) observed in the samples along with the previous study [28]. Starch LPLs were obtained by further extraction of rice starch with 0.8 mL of 75% n-propanol (*v/v*) at 100 °C for 2 h. The extracts thus obtained were subjected to the same LC–MS method to determine starch LPLs.

### 2.5. Total Starch, Amylose and Protein Content of Rice Flour

White rice flour was used for a total starch content (TSC) determination using a Megazyme total starch analysis kit (Megazyme Co., Ltd., Wicklow, Ireland). Samples were duplicated and fitted to a 96-well microplate following the manufacturer’s procedure (AACC Method 76.13). The amylose content was determined by iodometric estimation [29]. Protein content was determined by measuring the nitrogen content using a mass spectrometry method employing a Europa 20–20 isotope ratio mass spectrometer as described by Li et al. [29].

### 2.6. Starch Gelatinization Characterisation Using Differential Scanning Calorimetry and Swelling Power

Rice starch samples of 45 mg were homogenized in 90 mL water in duplicate and used to determine the starch thermal profiles of each line using a Differential Scanning Calorimeter (DSC 8000, PerkinElmer, Waltham, MA, USA). The procedure was conducted according to the published method and data were analyzed using the manufacturer software supplied with the instrument [28]. About 20 mg of starch was used for swelling power analysis following the method of Konik-Rose et al. [30]. The swelling power of each sample was calculated and presented as the ratio of the sedimented swollen weight to the initial dry weight of the starch.

### 2.7. Determination of Starch Molecular Structure Using FACE

The starch samples were debranched with isoamylase (Megazyme Ltd., Ireland). The sample preparation followed the published method with modifications [31]. The molecular structure of amylopectin was obtained by characterizing the chain-length distribution (CLD) by fluorescence-activated capillary electrophoresis (FACE) with a Laser-Induced Fluorescent detector (Agilent 7100 CE-LIF) at 488nm, and the data were analyzed using Agilent OpenLAB CDS ChemStation software (Agilent Technologies Australia Pty Ltd, Mulgrave, Victoria) [31].

### 2.8. Statistical Analysis

Statistical analyses were performed using *t*-test and Analysis of Variance of Genstat version 9 at the level of *p* < 0.05 and *p* < 0.01 for statistical significance.

## 3. Results

### 3.1. Genotyping and Single Grain Total Fatty Acid Profiling

The genotype of the *FAD2-1* RNAi backcross population progenies was determined by PCR markers and single-grain TFA profiling. Among 22 F_2_ progeny, four transgenic null sergeants (D5-17, D5-18, D5-20, and D5-22) were identified and thus considered as a homozygous *FAD2^WT^* genotype. The other 18 progeny carrying the RNAi construct were screened further by single-grain TFA profiling. The TFA composition of individual seeds of D5-1, D5-5, D5-7 and D5-8 consistently showed higher levels of oleic acid with approximately 20% C16:0, 48% C18:1 and 24% C18:2 in all the single-grain samples (Table 1). As no further segregation was observed in the F_4_ grains of D5-1, D5-5, D5-7 and D5-8, they were confirmed as a homozygous *FAD2^DR^* genotype and used for further analyses. The TFA of *FAD2^DR^* seeds contained a 12% decrease in C16:0, a 76% increase in C18:1 and a 41% decrease in C18:2 based on *FAD2^WT^* (Table 1). This was consistent with our previous study in which an increase in C18:1 and simultaneous reductions in the contents of C18:2 and C16:0 were observed in the seeds of *FAD2^DR^* relative to *FAD2^WT^* [26].

### 3.2. Characterisation of Non-Starch Lipids and Starch Lipids

**Figure 1:**
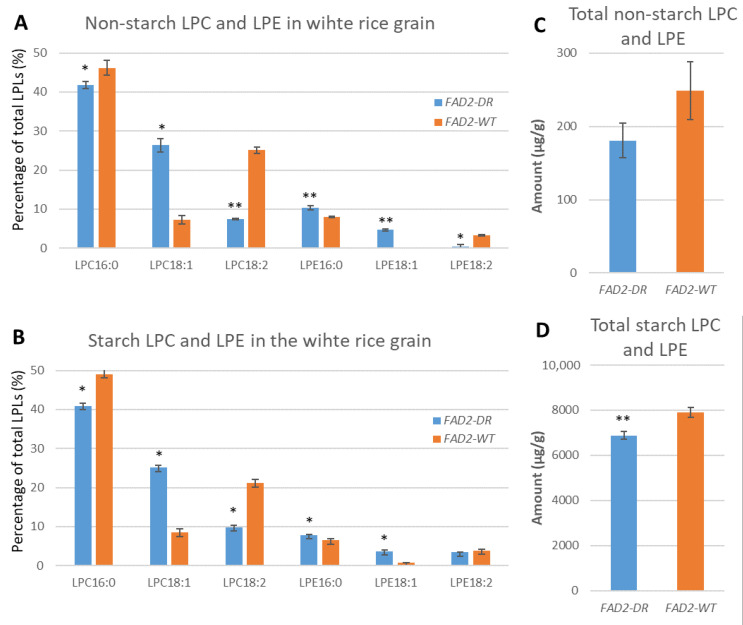
Fatty-acid composition of lysophospholipids in the white rice flour of *FAD2-1* RNAi backcross lines. (**A**,**B**) Comparison of the fatty-acid composition of LPC and LPE in the non-starch and starch lipids between *FAD2^DR^* and *FAD2^WT^*; (**C**,**D**) Comparison of total LPC and LPE in the non-starch and starch lipids between *FAD2^DR^* and *FAD2^WT^* flours. * and ** indicate the significance of difference at *p* <0.05 and *p* <0.01, respectively.

LPLs were reported to be involved in starch crystallization, which had a physicochemical impact on the starch properties of white rice [15,32]. Because LPC and LPE were the major LPLs of starch lipids associated with amylose in the rice endosperm [5], in this study we focused on the fatty-acid composition changes in LPC and LPE in particular of the *FAD2^DR^* endosperm. Starch lipids and non-starch lipids were separately extracted and analyzed using the LPC- and LPE-targeted LC-MS method [28]. The amount of total LPC and total LPE was 38- and 32-fold higher than for the non-starch counterparts in *FAD2^DR^* and *FAD2^WT^*, respectively (Figure 1 and Supplementary Table S1). In comparison with *FAD2^WT^*, the starch LPC/LPE and non-starch LPC/LPE in *FAD2^DR^* were reduced by 13% and 27%, respectively. As for the fatty-acid composition, LPCs and LPEs exhibited a similar profile but the amount of LPC was remarkably higher than LPE in both *FAD2^DR^* and *FAD2^WT^*. C16:0 was the most abundant in the LPC and LPE of both starch and non-starch lipids, accounting for about half of total fatty acids. C18:1 and C18:2 together comprised 30–40% of total fatty acids for LPC and LPE in *FAD2^DR^* and *FAD2^WT^*. The comparison between starch and non-starch fractions indicated the fatty-acid composition was almost comparable except for LPE18:2 in *FAD2^DR^*. In the starch lipids of *FAD2^DR^*, LPE18:2 was only slightly reduced, whereas in the non-starch lipids, LPE18:2 was barely discernible in *FAD2^DR^*. There was a clear consistency between the LPC and LPE derived from *FAD2^DR^* regardless of the lipid fraction (non-starch or starch): the proportion of C18:1 increased at the expense of C18:2. Interestingly, LPE16:0 increased slightly but decreased in the non-starch and starch LPCs of *FAD2^DR^*.

### 3.3. White Rice Flour Composition and Starch Properties

White rice flour was used to determine the main chemical composition of rice endosperm. *FAD2^DR^* showed a 2% increase in the TSC and a 2% decrease in the protein content compared with *FAD2^WT^* flours (Table 2). The amylose content was not statistically different between *FAD2^DR^* and *FAD2^WT^* flours (Table 2). Changes in starch thermal properties were observed through the variation in gelatinization temperatures (GT) and enthalpies (∆H) in the two primary peaks of the DSC curves [33]. Peak 1 indicated the thermal characteristic changes of starch gelatinization with a peak temperature at about 65 °C, and peak 2 represented ALC dissociation starting at just over 100 °C (^2^T_o_) (Table 3). The temperatures of peak 2 indicated the stability of the ALC, and ^2^∆H correlated with the amount of the ALC. The variation detected in the GTs and ∆H in peak 1 was not statistically different between *FAD2^DR^* and *FAD2^WT^*. In peak 2, ^2^T_p_ was higher but ^2^T_o_ was at the same temperature as *FAD2^WT^*. Despite the non-statistical significance, a nearly 29% increase in ^2^∆H was observed in *FAD2^DR^* (Table 3). In the swelling power test, the *FAD2^DR^* starch showed a significant reduction (13%) in comparison with *FAD2^WT^*.

### 3.4. Starch Molecular Structure Analyses

The overall CLD profiles of the debranched starch in both *FAD2^DR^* and *FAD2^WT^* grains exhibited a consistent pattern composed of about 20% ∑DP ≤10 and 90% ∑DP ≤24 glucan chains and a maximum number of glucan chains at DP12 (Supplementary Figure S1). The ratio (R_CL10/24_) of ∑DP ≤10 and ∑DP ≤24 in the CLD profile was almost identical for *FAD2^DR^* and *FAD2^WT^* starches. The only difference was shown by subtracting the CLD of *FAD2^WT^* from *FAD2^DR^*. However, the variation was insignificant with just over 0.4% at DP10 and even smaller changes observed at other chain lengths. The subtle changes in the starch molecular structure and composition of *FAD2^DR^* flour suggested the starch accumulation was not affected by the substantial reduction of FAD2 activity or increase in oleic acid in transgenic grains. The variation of starch structure and grain composition of white rice flour between *FAD2^DR^* and *FAD2^WT^* was considered negligible, not significant enough to affect starch properties. Therefore, the alteration of *FAD2^DR^* starch property can be primarily attributed to fatty-acid composition changes.

## 4. Discussion

### 4.1. Indication of Lipid Accumulation in White Rice Grain

According to the fatty-acid biosynthesis previously described, PC18:1 and PE18:1 are desaturated by FAD2 to produce PC18:2 and PE18:2 at the ER membrane [34]. PC18:1 and PE18:2 can be processed to produce DAG and be further converted into TAG or deacylated to form LPC18:1 and LPE18:2 [35]. Our observation that the fatty-acid composition is consistent with a high C18:1 content between LPC and LPE in the endosperm and TFA (mainly TAG) composition at whole seed level of *FAD2^DR^* suggests that the precursor PC18:1 and PE18:1 accumulated with a low level of PC18:2 and PE18:2 produced because of low FAD2 activity. However, the fatty-acid composition of PC and PE needs to be investigated further. Within the endosperm of *FAD2^DR^* grain, the fatty-acid composition of LPC and LPE are also consistent between starch and non-starch fractions, which are considered as lipids contained in the amyloplast and the ER compartment, respectively. This can be possibly attributed to the extensive trafficking of acyl groups between ER and the plastid, which has been regarded as a key aspect of the eukaryotic pathway [24]. In maize endosperm, a proposed model of the ER–amyloplast lipid trafficking suggests that LPC derived from PC is imported from the ER to the amyloplast [16]. LPE is a minor form of trafficking lipids and its translocation would presumably follow a similar fashion to that of LPC. Nevertheless, the current understanding of lipid biosynthesis in amyloplast and overall endosperm lipid metabolism is rather limited and only based on what is known about the biosynthesis of chloroplast lipids in photosynthetic organs.

LPC and LPE produced through the eukaryotic pathway exhibit different *sn* positions depending on the carbon chain length of the fatty acids [16,36]. Accordingly, the C16:0 is at the *sn*-1 position in rice starch LPLs, while C18:1 and C18:2 may occupy the *sn-2* position of the glycerol backbone. The linear steric orientation of LPC 16:0 and LPE 16:0 molecules may be more efficient for the ALC formation, while LPC/LPE C18:1 and C18:2 form ALCs in a less efficient manner. Consequently, a greater amount of LPC 16:0 and LPE 16:0 is trapped inside the starch granule during endosperm development than 18-carbon LPC and LPE (C18:1 and C18:2). In contrast, a much lower proportion of C16:0 was found in the storage lipids at the whole seed level than in the endosperm because C16:0 is elongated to C18:0 for further desaturation to C18:1 and then C18:2 by stearoyl–ACP fatty acid desaturase (SAD) in the plastid and FAD2 in the ER, respectively [24]. The pleiotropic effect of the reduction in LPC16:0 and LPE16:0 of starch LPLs in *FAD2^DR^* endosperm again supports the theory that LPC and LPE originate from ER-derived PC and PE via ER–amyloplast lipid trafficking [16]. It was also explained in the previous study that a reduction in palmitic acid content in the *FAD2^DR^* could be compensatory for maintaining sufficient C18:2 content at the whole-seed level [26].

### 4.2. Formation of Amylose–Lipid Complex and Functional Properties of High-Oleic Rice Flour

Adding lipids to starch with stirring and heating at 65–75 °C in excess water can form ALCs in an exothermic manner during starch gelatinisation [37]. The formation of ALCs increases the starch gelatinisation temperature and decreases starch solubility and swelling power [38]. Some reports suggest that a lipid with an acyl chain length of 14 carbons is efficient for complexation, while others find 16 or 18 carbons to be the preferred acyl chain length [7]. Even though the amount of total starch LPLs was reduced, and possibly the same for the ALC in the *FAD2^DR^* starch for an unknown reason, it still showed restricted gelatinisation as indicated by the reduction in the swelling-power test, which could be due to an increase in C18:1 and a decrease in C16:0 and C18:2. This is because the crystal structure of rice starch with C18:1 is more ordered than C18:2, and the ALC formed by amylose and C18:1 is more stable compared to C18:2 [32]. In contrast to the efficiency of forming ALCs, a fatty acid with a long carbon chain is more stable in maintaining the ALC structure [7].

In the heat moisture treatment of food processing, fatty acids are blended with starch, and it is believed they can diffuse into the interspace of starch granules and enter the amylose cavity to form ALCs and enhance the crystalline structure with a higher ordering degree [32]. In an early study on ALCs, 10% (*w/w*) C18:1 or C18:2 was added to maize starch, which was cooked [14]. Although there was no remarkable temperature change in starch gelatinisation between adding these two types of fatty acids, the increases in ^2^T_p_ and ^2^T_e_ together with a slight increase in ^2^∆*H* of the ALC were detected in the starch with the C18:1 addition [14]. In our current study, there was similarly no difference in starch GTs, but ^2^T_p_ and ^2^∆H of the ALC were higher in *FAD2^DR^* than *FAD2^WT^*. This evidence indicated high stability of ALCs with C18:1 required a higher temperature to dissociate than the ALCs with a high C18:2 in *FAD2^WT^*. The dissociation temperatures of the ALC have been reported to increase along with the length of the carbon chains but decrease with the increasing number of fatty-acid double bonds [19,20,21,22]. This suggests the double bonds in the unsaturated fatty-acid molecules may impair or affect the stability of ALCs. The increased GTs and ^2^∆H of the ALC is a net effect of increased C18:1 and decreases of C16:0 and C18:2 in starch LPLs of the *FAD2^DR^* grain.

## 5. Conclusions

The fatty acid composition changes (C18:1 increased at the expense of C18:2) in LPCs and LPEs in non-starch and starch fractions of the *FAD2^DR^* rice grain were generally in agreement with the TFA changes in the whole grain. The *FAD2^DR^* starch, containing the ALC formed by amylose and LPC/LPE enriched with C18:1, required higher energy to disassociate and exhibit reduced starch swelling power than that of *FAD2^WT^*. LPCs and LPEs in the starch lipid fraction were produced from ER-derived PCs and PEs. The study provides an insight into the mechanism of lipid accumulation in a cereal endosperm and the functional properties of ALCs due to the genetic modification of fatty-acid composition.

## Figures and Tables

**Table 1:** Total fatty acid composition of brown rice flour of the selected *FAD2-1* RNAi backcross lines.

Genotype	C14:0 (%)	C16:0 (%)	C18:0 (%)	C18:1 (%)	C18:2 (%)
*FAD2^DR^*	0.49 (0.02) **	20.23 (0.58) **	3.32 (0.25)	48.07 (1.62) **	24.13 (2.16) **
*FAD2^WT^*	1.10 (0.10)	22.90 (0.26)	3.39 (0.15)	27.26 (0.92)	40.82 (0.91)

Mean values are the average of three independent lines of each genotype. STD are shown in the brackets. ** indicates the significance of difference at *p* < 0.01.

**Table 2:** White rice flour composition of the selected *FAD2-1* RNAi backcross lines.

Sample	Total Starch (%)	Amylose (%)	Protein (%)
*FAD2^DR^*	74.47 (1.28) **	14.63 (4.11)	7.41 (0.81) *
*FAD2^WT^*	72.28 (1.29)	12.00 (3.16)	9.41 (0.68)

Mean values are the average of three independent lines of each genotype. Standard deviations are shown in the brackets. * and ** indicate the significance of difference at *p* < 0.05 and *p* < 0.01, respectively.

**Table 3:** Rice grain starch thermal characteristics of *FAD2^DR^* and *FAD2^WT^* determined by DSC.

Genotype	Peak 1				Peak 2				Swelling Power
	^1^To (°C)	^1^Tp (°C)	^1^Te (°C)	^1^∆H (J/g)	^2^To (°C)	^2^Tp (°C)	^2^Te (°C)	^2^∆H (J/g)	
*FAD2^DR^*	55.45 (1.36)	65.44 (2.08)	83.41 (2.06)	4.78 (0.48)	100.07 (2.49)	110.75 (1.32) **	118.42 (1. 89)	0.49 (0.04)	11.84 (0.25) **
*AD2^WT^*	55.36 (1.54)	64.02 (0.52)	81.88 (1.69)	4.42 (0.51)	100.26 (1.03)	109.18 (0.86)	116.97 (1.10)	0.38 (0.08)	13.57 (0.28)

^1^To, ^1^Tp and ^1^Te are onset, peak and end gelatinization temperatures, and ^1^ΔH is the gelatinization enthalpy at the amylopectin gelatinization peak. ^2^To, ^2^Tp, and 2Te are onset, peak and end dissociation temperatures of amylose–lipid complexes, and ^2^ΔH is the dissociation enthalpy at the dissociation peak of amylose-lipid complexes. Mean values are the average of the corresponding three lines of *FAD2^DR^* and *FAD2^WT^*, respectively. ** indicates the significance of difference at *p* < 0.05 and *p* < 0.01, respectively. STD are shown in the brackets.

## Supplementary Materials

The following are available online at https://www.mdpi.com/article/10.3390/foods10061169/s1, Figure S1: Comparison of chain length distribution of debranched starch of *OsFAD2-1* RNAi backcross lines. Table S1: Fatty-acid composition of lysophospholipids in the white rice flour of *OsFAD2-1* RNAi backcross lines.
