# Supplementary material for: Down-Regulation of FAD2-1 Gene Expression Alters Lysophospholipid Composition in the Endosperm of Rice Grain and Influences Starch Properties
Source: Foods. 2021 May 23;10(6):1169. doi: 10.3390/foods10061169 (PMC8224701; doi:10.3390/foods10061169)

Supplementary Table S1. Fatty acid composition of lysophospholipids in the white rice flour of *OsFAD2-1* RNAi backcross lines.

LPL fraction	Genotype	LPC 14:0 (%)	LPC 16:0(%)	LPC 18:0 (%)	LPC 18:1(%)	LPC 18:2(%)	LPC 18:3(%)	Total LPC (%)	LPE 14:0 (%)	LPE 16:0 (%)	LPE 18:0 (%)	LPE 18:1 (%)	LPE 18:2(%)	LPE 18:3 (%)	Total LPE (%)	Total amount (ug/g)
Starch LPL	*FAD2*^{DR}	1.94 (0.1592)	40.94 (0.6386)	5.72 (0.2169)	25.08 (0.6921)	9.85 (0.4353)	0.46 (0.0370)	84.00 (0.3178)	0.26 (0.0172)	7.91 (0.1105)	0.49 (0.0764)	3.74 (0.1476)	3.44 (0.0567)	0.17 (0.0065)	16.00 (3.1780)	6881 (162.4)
	FAD2^{WT}	4.39 (0.5237)	49.05 (1.2991)	4.46 (0.2953)	8.51 (0.8810)	21.18 (0.8355)	0.43 (0.0223)	88.02 (0.5701)	0.39 (0.0634)	6.51 (0.3422)	0.34 (0.0194)	0.78 (0.0452)	3.87 (0.3203)	0.10 (0.0130)	11.98 (0.5701)	7900 (218.8)
	P value	0.001	< 0.001	0.004	< 0.001	< 0.001	0.246	< 0.001	0.024	0.003	0.029	< 0.001	0.083	< 0.001	< 0.001	0.003
Non-Starch LPL	*FAD2*^{DR}	2.53 (0.0573)	41.81 (0.9130)	6.42 (0.3409)	26.37 (1.7510)	7.48 (0.2491)	ND	84.61 (0.5988)	ND	10.35 (0.5495)	ND	4.67 (0.2424)	0.37 (0.6365)	ND	15.39 (0.5988)	180.7 (23.46)
	FAD2^{WT}	5.41 (0.3113)	46.19 (1.8490)	4.77 (0.5435)	7.22 (1.0760)	25.13 (0.7808)	ND	88.65 (0.0734)	ND	8.03 (0.1673)	ND	ND	3.31 (0.1447)	ND	11.35 (0.0734)	248.5 (39.56)
	P value	<0.001	0.021	0.010	< 0.001	< 0.001	NA	0.007	NA	0.002	NA	< 0.001	0.001	NA	0.007	0.063

LPC is lysophosphatidylcholine, and LPE is lysophosphatidylethanolamine. ND is not detected. NA is not available. Mean values are the average of three independent lines of each genotype. STD are shown in the brackets.

Supplementary Figure S1. Comparison of chain length distribution of debranched starch of *OsFAD2-1* RNAi backcross lines. Starches from three independent line of each genotype were used for the analysis with two replicates for each sample. Error bars indicate the standard deviation. Asterisks indicate differences at the statistical level, $P < 0.5$. A, CLD profiles of *FAD2*^{DR} and *FAD2*^{WT} lines. B, Differences in the CLD profiles of amylopectin between *FAD2*^{DR} and *FAD2*^{WT} lines. C, Comparison of CLD proportions in short chains (∑DP≤10), intermediate chains (∑DP≤24) and their ratio ($R_{CL10/24}$).

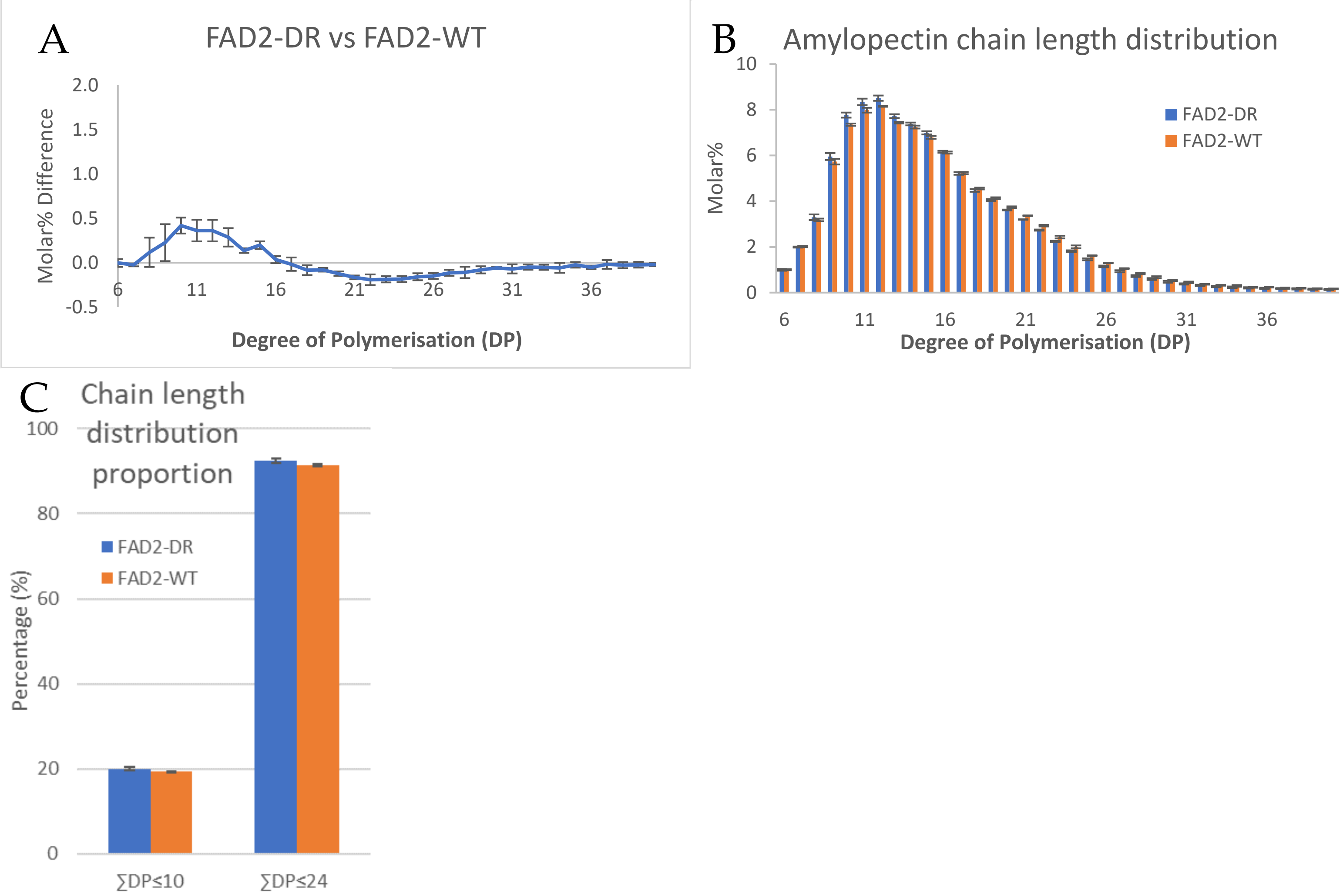

Supplement: Supplementary file 1 [file foods-10-01169-s001.zip › foods-1191931-supplementary.pdf]
